# Freshwater diatoms in the Democratic Republic of the Congo: a historical overview of the research and publications

**DOI:** 10.3897/phytokeys.136.47386

**Published:** 2019-12-23

**Authors:** Christine Cocquyt, Edit Lokele Ndjombo, Simon Tutu Tsamemba, Hippolyte Nshimba Seya wa Malale

**Affiliations:** 1 Research Department, Meise Botanic Garden, Nieuwelaan 38, 1860, Meise, Belgium; 2 Institut Facultaire des Sciences Agronomiques de Yangambi, Kisangani, DR Congo; 3 Faculté de Gestion des Ressources Naturelles et Renouvelables, Université de Kisangani, DR Congo; 4 Faculté des Sciences, Université de Kisangani, DR Congo

**Keywords:** Algae, Bacillariophyta, Congo basin, tropical Africa, Zaire

## Abstract

An overview of the diatom research in the DR Congo is given based on literature data starting in 1938 with the work of Zanon and excluding the East African Lakes as these were already discussed in previous papers. For each literature record the diatom genera mentioned are presented as well as all diatom taxa described from the Congo as new. In total, 106 new taxa were documented, of which *Nitzschia* with 40 taxa is far the most important genus followed by *Navicula* s.l. and *Pinnularia* and with 15 and 13 taxa respectively. Particular attention was paid to the local research of students found in unpublished theses at bachelor, licentiate, master and PhD level. Diatom records in these works are almost all restricted to genus level, although in the last decade an attempt to delimit species can be observed. This accompanies the renewed taxonomic interest in the Congo basin during the last decade. Renewed taxonomic interest can also be seen in the genera: the first period being situated during the lumping period, while more recent works follow the current taxonomic classification, for example *Navicula* s.l. versus *Navicula*, *Cavinula*, *Craticula*, *Diadesmis*, *Geissleria*, *Humidophila*, *Luticola*, *etc*.

## Introduction

In the Democratic Republic of the Congo (DR Congo), research in the field of plant biology mostly concerned the study of terrestrial forest ecosystems (Anonymous 2012; Wasseige et al. 2014), while the interest given to the aquatic environments was mainly limited to ichthyology and fisheries (e.g. Chapman 2001; Paugy et al. 2011; Snoeks et al. 2011), fish being an important source of protein for local populations. Only a few publications are available in the field of microscopic algae, and diatoms in particular, from inland aquatic environments in DR Congo.

Early publications on freshwater algae in tropical Africa focused on the great lakes of the Albertine rift: Malawi (Nyassa/Nyasa), Tanganyika (Tanganika) and Victoria (Victoria Nyanzae) (e.g. Müller 1904a, b, 1905, 1910; West 1907). A review of the studies that have been carried out on these large lakes and the lentic and lotic ecosystems of East Africa is given by Cocquyt (2006) and Taylor and Cocquyt (2015).

The present paper aims to give an overview of the research that has been conducted in the DR Congo or that has investigated Congolese material, not only found in international publications, but also by means of local publications as well as unpublished theses at different levels (bachelor, licentiate, master and PhD).

## Material and methods

Initially international publications on algae, and more specifically on the diatoms, of DR Congo (formerly Belgian Congo between 1908 and 1960, and the Republic of Zaire between 1971 and 1997) were searched for on the Web of Science and in the available international literature. Subsequently, inquiries were made regarding papers that were published locally in journals of the different universities in DR Congo and in final reports of national and international projects. The last step was to retrieve all theses, PhD level and other dissertations (licentiate, bachelor and master level) from universities and scientific institutions in DR Congo. Licentiate is an academic degree below that of a PhD, used in Belgium (and the DR Congo), and obtained after a university study of 4 to 5 years. In the Bachelor-Master structure, it is the degree that corresponds to (almost) a Master.

## Results and discussion

A brief overview follows on the diatom research conducted on materials collected in DR Congo, including the former Congo and the Republic of Zaire, as found in mainstream literature, thus taking into consideration only published work accessible to the international scientific community.

The diatoms of Lake Kivu and its surroundings were first documented by Zanon (1938). From this region he reported 263 taxa belonging to 33 genera. Of these taxa, 16 were new to science of which 9 were *Pinnularia* Ehrenberg, the others belonging to the genera *Cocconeis* Ehrenberg, *Cymbella* C. Agardh, *Eunotia* Ehrenberg, *Neidium* Pfitzer and *Synedra* Ehrenberg (for details see Table 1). However, we must point out that most of the genera names mentioned in this paper are in the broad sense (sensu lato) as they very probably combine several genera after the changes initiated in recent diatom classification (Round et al. 1990, and subsequent later taxonomic publications). These systematic changes were initially based on knowledge acquired through what were relatively new technologies at the time such as the scanning electron microscope and more recently molecular analyses.

**Table 1.** New diatom taxa described from DR Congo, their references, geography, habitat and possible synonyms.

Taxon	Publication	Page	Plate	fig.	Province	Region	Waterbody	Synonym
Achnanthes atomus var. congolensis Hustedt***	Hustedt 1949	74–75	2	35, 36	North-Kivu	Bugazia	Lake Edward	*Achnanthes congolensis* Hustedt
*Achnanthes pseudogrimmei* Cholnoky*	Cholnoky 1970	11–2	1	1–3	Zambia	10 km from Kansenga	Lake Chali	
*Amphora thermalis* Hustedt*	Hustedt 1949	111–112	11	1–3	North-Kivu	May-ya-Moto	hot springs	*Halamphora thermalis* (Hustedt) Levkov
*Amphora submontana* Hustedt*	Hustedt 1949	112–113	11	4	North-Kivu	Butembo	Mosenda river mouth in Lake Edward	*Halamphora submontana* (Hustedt) Levkov
*Cavinula lilandae* Cocquyt, M. de Haan & J. C. Taylor	Cocquyt et al. 2013	158		2–11, 16–21	Tshopo	Lilanda	Baombe stream	
*Cocconeis citrina* Zanon*	Zanon 1938	598		6	South-Kivu	Kivu	stream	*Cocconeis vitellina* Schoeman
*Cocconeis scaettae* Zanon*	Zanon 1938	598–599		7	South-Kivu	Kivu	stream	
*Coscinodiscus antiquus* [var. minor] f. bananaensis Kufferath*	Kufferath 1956	43	3	1	Bas-Congo	Banana	creek near the ocean	
*Craspedodiscus minutus* Kufferath*	Kufferath 1956	45	3	2	Bas-Congo	Banana	creek near the ocean	
*Cymbella naviculoides* Hustedt*	Hustedt 1949	113–114	10	9–13	North-Kivu	Karisimbi	pond at 3800 m	*Encyonopsis naviculoides* (Hustedt) Krammer
Cymbella norvegica var. parva Zanon*	Zanon 1938	605–606		38	North-Kivu	Karisimbi	puddle at 3900 m	
*Cymbellonitzschia cataractorum* Kufferath*	Kufferath 1957	20–21		41	Rwanda	Bugarama	rapids on the Rusizi river	
*Eunotia damasii* Hustedt*	Hustedt 1949	67–68	3	1–12	North-Kivu	Karisimbi	crater lake at 3800 m	
*Eunotia fuseyi* J.C. Taylor & Cocquyt	Taylor et al. 2016a	305		11–14	Tshopo	Yangubu	Lobaye river	*Eunotia pierrefuseyi* (J.C. Taylor & Cocquyt) J.C. Taylor & Cocquyt
*Eunotia leonardii* J.C. Taylor & Cocquyt	Taylor et al. 2016a	295		6–10	Tshopo	Yangubu	Lobaye river	
*Eunotia montana* Hustedt*	Hustedt 1949	66–67	3	13–23	North-Kivu	Gando	lake	
*Eunotia rudis* Cocquyt & M. de Haan	Cocquyt et al. 2016	75–76		2–24	Tshopo	Yangambi	Libongo river	
*Eunotia scaettae* Zanon*	Zanon 1938	595–596		3	North-Kivu	Karisimbi	puddle at 2000 m	
*Eunotia pseudoflexuosa* Hustedt	Hustedt 1949	71–72	2	16–18	North-Kivu	Karisimbi	crater lake	
*Fragilaria africana* Hustedt*	Hustedt 1949	62	2	29–34	North-Kivu	Bugazia, Kamande	Lake Edward	*Staurosirella africana* (Hustedt) D.M. Williams & Round
*Geissleria lubiluensis* Cocquyt & Lokele	Cocquyt and Lokele 2019	243–244		1–4, 6–17	Tshopo	Yangambi	Lubilu river	
*Gomphonema aequatoriale* Hustedt*	Hustedt 1949	119–120	10	6–8	North-Kivu	Kamande	Lake Edward	
*Gomphonema constrictum* [var. capitata] f. bipunctata Kufferath*	Kufferath 1957	30–31		38	Rwanda	Bugarama	rapids on the Rusizi river	
*Gomphonema grande* B. Karthick, Kociolek, J.C. Taylor & Cocquyt	Karthick et al. 2016	188		1–24	Tshopo	Yangubu	Lomami river	
*Gomphonema zairense* Compère*	Compère 1995	32		1–14	Tshopo	Kisangani	Tshopo waterfalls	
*Hantzschia ruziziensis* Kufferath*	Kufferath 1957	34		53	Rwanda	Bugarama	rapids on the Rusizi river	
*Hantzschia uncinata* Kufferath*	Kufferath 1957	34–35		45	Rwanda	Bugarama	rapids on the Rusizi river	
*Melosira mareei* Kufferath*	Kufferath 1956	42	2	5	Bas-Congo	Banana	ocean – brackish-water	
*Navicula barbarica* Hustedt*	Hustedt 1949	97	4	14–17	North-Kivu	Kamande	Lake Edward, Mosenda river mouth	
*Navicula congolensis* Hustedt*	Hustedt 1949	86	4	23, 24	North-Kivu	Gando	pond	
*Navicula dartevellei* Kufferath*	Kufferath 1957	23		25	Rwanda	Bugarama	rapids on the Rusizi river	
*Navicula faceta* Hustedt*	Hustedt 1949	88	4	25, 26	North-Kivu	Gando	lake	
*Navicula finitima* Hustedt*	Hustedt 1949	90	4	29, 30	North-Kivu	Kamande	creek	
*Navicula marlieri* Kufferath*	Kufferath 1957	23–24		26	Rwanda	Bugarama	rapids on the Rusizi river	
*Navicula molestiformis* Hustedt*	Hustedt 1949	86–87	5	9	North-Kivu	Kamande	Lakes Edward	*Craticula molestiformis* (Hustedt) Mayama
*Navicula muraliformis* Hustedt*	Hustedt 1949	85–86	4	31, 32	North-Kivu	Karisimbi	crater lake	*Mayamaea muraliformis* (Hustedt) Lange-Bertalot
*Navicula muticoides* Hustedt*	Hustedt 1949	82	4	33–36	North-Kivu		Lakes Kivu (Bera), Ndalaga	*Luticola muticoides* (Hustedt) D.G. Mann
*Navicula nielsfogedii* J.C. Taylor & Cocquyt	Taylor et al. 2016b	202		1–22, 34–51	Tshopo	Yangubu	Lomami river	
Navicula subcontenta var. africana Hustedt*	Hustedt 1949	85	4	27, 28	North-Kivu	Kasinga-Channel (Uganda)		
*Navicula submolesta* Hustedt*	Hustedt 1949	86	5	16–18	North-Kivu	Gando	puddle	*Craticula submolesta* (Hustedt) Lange-Bertalot
*Navicula zanonii* Hustedt*	Hustedt 1949	92–93	5	1–5	North-Kivu	Bougeria, Bugazia, Kamande	Lakes Edward, Kivu	
Neidium iridis var. parallela Zanon*	Zanon 1938	619		1	North-Kivu	Karisimbi	puddle at 3900 m	
*Nitzschia accommodata* Hustedt*	Hustedt 1949	139	12	27–31, 34, 35	North-Kivu	Ngoma	Lake Kivu	
*Nitzschia adapta* Hustedt*	Hustedt 1949	135	12	3–6	North-Kivu	Kamande	Lakes Edward, Kibuga, Ndalaga	
*Nitzschia aequalis* Hustedt*	Hustedt 1949	135–136	12	7, 8	North-Kivu	Bugazia	Lake Edward	
*Nitzschia amphioxoides* Hustedt*	Hustedt 1949	140	13	65–72	North-Kivu	Kamande, Bugazia	Lake Edward	
Nitzschia bacata f. linearis Hustedt*	Hustedt 1949	149	13	17–20	North-Kivu	Kamande	Lake Edward	
*Nitzschia baculumata* Kufferath*	Kufferath 1956	55	7	3	Bas-Congo	Banana	ocean – brackish-water	
*Nitzschia biconicacuta* Kufferath*	Kufferath 1957	36–37		57	Rwanda	Bugarama	rapids on the Rusizi river	
Nitzschia caparti*	Kufferath 1948	8		17	Equateur	Makanza (Nouvelle-Anvers)	Congo River	
*Nitzschia confinis* Hustedt*	Hustedt 1949	145	11, 13	49–54, 84–90	North-Kivu, South-Kivu	Ngoma, Keshero, Kishushu, Nyamirundi	Lakes Kivu, Ndalaga	
*Nitzschia congolensis* Hustedt*	Hustedt 1949	134	12	15, 16	North-Kivu	Kamande, Vitshumbi	Lake Edward	
*Nitzschia consummata* Hustedt*	Hustedt 1949	134–135	12	1, 2	North-Kivu	Semliki	Lake Edward	
*Nitzschia curvirectangularis* Kufferath*	Kufferath 1956	55	5	8	Bas-Congo	Banana	ocean – brackish-water	
*Nitzschia diserta* Hustedt*	Hustedt 1949	139	12	32, 33	South-Kivu	Nyamirundi	Lake Kivu	
*Nitzschia elliptica* Hustedt	Hustedt 1949	148–149	13	32–34	North-Kivu	May-ya-Moto	hot springs	
*Nitzschia epiphyticoides* Hustedt*	Hustedt 1949	144–145	13	48–55	North-Kivu	Semliki	Lakes Edward, Kivu	
*Nitzschia fusulata* Kufferath*	Kufferath 1956	56	5	9	Bas-Congo	Banana	creek near the ocean	
*Nitzschia hexagonata* Kufferath*	Kufferath 1956	56	2	10A, B	Bas-Congo	Banana	creek near the ocean	
Nitzschia hexagonata f. minutissima Kufferath*	Kufferath 1956	56	1, 2	12C, 9, 12A–E	Bas-Congo	Banana	ocean – brackish-water	
*Nitzschia inflata* Kufferath*	Kufferath 1957	38		64	Rwanda	Bugarama	rapids on the Rusizi river	
*Nitzschia intermissa* Hustedt*	Hustedt 1949	136	12	11–14	North-Kivu, South-Kivu	Kamande, Katana	Lake Edward, Machusa-waterfall near Lake Kivu	
*Nitzschia latens* Hustedt*	Hustedt 1949	148	13	30–31	North-Kivu	May-ya-Moto	hot springs	
*Nitzschia mammalifera* Kufferath*	Kufferath 1956	57	6	4, 5	Bas-Congo	Banana	ocean – brackish-water	
*Nitzschia mareei* Kufferath*	Kufferath 1956	58	5	11	Bas-Congo	Banana	creek near the ocean	
*Nitzschia mediocris* Hustedt*	Hustedt 1949	149	13	21–24	South-Kivu	Nyamirundi	Lake Kivu	
*Nitzschia microsicula* Kufferath*	Kufferath 1956	58	5	4	Bas-Congo	Banana	creek near the ocean	
*Nitzschia obsidialis* Hustedt*	Hustedt 1949	148	13	25	North-Kivu	Kamande	Lake Edward	
*Nitzschia obsoleta* Hustedt*	Hustedt 1949	146	13	94–99	North-Kivu	Kamande, Bugazia	Lake Edward	
*Nitzschia ogivalis* Kufferath*	Kufferath 1957	40–41		56	Rwanda	Bugarama	rapids on the Rusizi river	
Nitzschia palea var. tropica Hustedt*	Hustedt 1949	147	13	26–29	North-Kivu	Kamande, Bugazia, Gando	Lakes Edward, Kivu, Kibuga, Ndalaga ponds	
*Nitzschia pseudopectinalis* Kufferath*	Kufferath 1957	42–43		73	Rwanda	Bugarama	rapids on the Rusizi river	
*Nitzschia rectangulata* Kufferath*	Kufferath 1957	43		70a, b	Rwanda	Bugarama	rapids on the Rusizi river	
*Nitzschia robusta* Hustedt*	Hustedt 1949	141	13	35–38	South-Kivu	Katana	Machusa-waterfall near Lake Kivu	
*Nitzschia spiculoides* Hustedt*	Hustedt 1949	151	13	5–6	North-Kivu	Semliki	Lake Edward	
*Nitzschia spiculum* Hustedt*	Hustedt 1949	136	13	1–4	North-Kivu, South-Kivu	Kasinga-Channel (Uganda), Bugazia, Kamande, Katana	Lake Edward, Machusa-waterfall near Lake Kivu	
*Nitzschia spirilliformis* Kufferath*	Kufferath 1956	58	6, 7	3, 1	Bas-Congo	Banana	ocean – brackish-water	
*Nitzschia stricta* Hustedt*	Hustedt 1949	136	12	9, 10	North-Kivu	Kamande	Lake Edward	
*Nitzschia subcommunis* Hustedt*	Hustedt 1949	146	11, 13	55–58, 101–106	South-Kivu, North-Kivu	Katana	Machusa-waterfall near Lake Kivu, Lakes Kibuga, Ndalaga	
*Nitzschia tarda* Hustedt*	Hustedt 1949	138–139	12	24, 25	North-Kivu	Kamande	Lake Edward	
*Nitzschia tropica* Hustedt*	Hustedt 1949	147	11	34–48	North-Kivu, South-Kivu	Kasinga-Channel (Uganda), Nyamirundi, Katana	Lakes Kibuga, Ndalaga, Lake Kivu, Machusa-waterfall	
*Nitzschia umbilicata* Hustedt*	Hustedt 1949	129–130	11	65	North-Kivu		Lake Kibuga	*Tryblionella umbilicata* (Hustedt) D.G. Mann
Pinnularia alpina var. parallela Zanon*	Zanon 1938	642–643		29	North-Kivu	Karisimbi	puddle at 3900 m	
Pinnularia borealis var. congolensis Zanon*	Zanon 1938	641		27	North-Kivu	Karisimbi	puddle at 3900 m	*Pinnularia congolensis* (Zanon) Cholnoky
*Pinnularia congolensi*s Zanon**	Zanon 1938	545, 571			North-Kivu	Karisimbi, Nanindhja	puddle at 3900 m, puddle at 2000 m	
*Pinnularia fusiformis* Zanon*	Zanon 1938	645–646		24	North-Kivu	Nanindhja	puddle at 2000 m	
Pinnularia lata var. biconstricta Zanon*	Zanon 1938	642		28	North-Kivu	Karisimbi	crater pond at 3900 m	
Pinnularia lata var. constricta Zanon*	Zanon 1938	642		30	Uganda	Mufumbiru	peat bog at 2160 m	Pinnularia borealis var constric*t*a (Zanon) Cholnoky
Pinnularia lata var. media Zanon*	Zanon 1938	643		25	North-Kivu	Karisimbi	puddle/crater pond at 3900 m	
*Pinnularia lineolata* Zanon*	Zanon 1938	647–648		23	North-Kivu	Nanindhja	puddle at 2000 m	
*Pinnularia scaettae* Zanon*	Zanon 1938	648		21	North-Kivu, South-Kivu	Karisimbi, Kahuzi	crater pond at 3950 m/ Bambu mud	
Pinnularia scaettae var. krasskei Zanon*	Zanon 1938	648		22	North-Kivu, South-Kivu	Karisimbi, Kahuzi	puddle at 3900 m/ Bambu mud	
*Pinnularia symoensii* Cholnoky*	Cholnoky 1970	51	2	4	Zambia	Lake Bangweolo	Tushingo Channel	
*Pinnularia tropica* Hustedt	Hustedt 1949	108–109	7	1–12	North-Kivu	Karisimbi	crater pond, pond at 3000 m	
*Pinnularia valida* Hustedt*	Hustedt 1949	106	6	22	North-Kivu	Karisimbi	pond at 3000 m	
*Pseudo-eunotia ruziziensis* Kufferath*	Kufferath 1957	19–20		40	Rwanda	Bugarama	rapids on the Rusizi river	
*Stauroneis subobtusa* Hustedt*	Hustedt 1949	80	5	25	Kivu		Lake Kivu (Bera)	
*Stauroneis zairensis* Compère*	Compère et al. 1989	224		2–6, 8–13		Kinshasa	Fish pond	
*Stephanodiscus damasii* Hustedt*	Hustedt 1949	57–58	1	2–5	North-Kivu	Simliki, Bugazia, Pili-Pili	Lakes Edward, Kivu Ndalaga	*Cyclostephanos damasii* (Hustedt) Stoermer & Håkansson
*Surirella congolensis* Cocquyt & J.C. Taylor	Cocquyt and Taylor 2015	8		6–9	Tshopo	Yangubu	Lomami river	*Iconella congolensis* (Cocquyt & J.C. Taylor) Cocquyt & J.C. Taylor
Surirella cuspidata f. constricta Hustedt*	Hustedt 1949	155	15	11	North-Kivu	Karisimbi, Gando	crater pond pond	
*Surirella ebalensis* Cocquyt & J.C. Taylor	Cocquyt and Taylor 2015	2		1–5	Tshopo	Yangubu	Lomami river	*Iconella ebalensis* (Cocquyt & J.C. Taylor) Cocquyt & J.C. Taylor
*Surirella propinqua* Hustedt*	Hustedt 1949	153	14	5–6	North-Kivu	Karisimbi	lake	*Iconella propinqua* (Hustedt) Cocquyt & R. Jahn
*Surirella symoensii* Cholnoky*	Cholnoky 1970	56	2	8	Zambia	Bwalya Mponda	Lake Chali	
*Synedra bananaensis* Kufferath*	Kufferath 1956	49	3	8	Bas-Congo	Banana	ocean – brackish-water	
*Synedra famelica var. enflata* Zanon*	Zanon 1938	586–587		11	North-Kivu, Uganda	Karisimbi, Mufumbiru, Nya-Mwindhja	puddle at 3900 m, peat bog at 2160 m, puddle at 1500 m	Fragilaria strangulata f. inflata (Zanon) Hustedt
*Synedra strangulata* Zanon*	Zanon 1938	587		14	North-Kivu	Karisimbi	puddle at 3900 m	*Fragilariforma strangulata* (Zanon) D.M. Williams & Round

* : taxon status uncertain ‘(unassessed) in DiatomBase (30 September 2019);

**: taxon not found in DiatomBase (30 September 2019);

***: taxon status unaccepted (synonym) in DiatomBase (30 September 2019).

Zanon (1938) described Pinnularia borealis var. congolensis Zanon (on page 641) with a drawing (fig. 27) from a puddle on the Karisimbi volcano in the region Lake Kivu and mentioned this taxon name also (on page 545) in his species list of the diatoms from the region of Lake Kivu. However, in the same publication Zanon (1938) wrote Pinnularia borealis var. africana v. n. (on page 556) and *Pinnularia congolensis* n. sp. (on page 571) and in the species list he mentioned sample nr 5 from a puddle on the Karisimbi volcano and sample nr 21 from a puddle from Nanindhja respectively. However, no description is given for these two taxa, consequently both names have to be considered nomina nuda and therefore invalidly published. Two decades later Cholnoky (1957) elevated the validly described Pinnularia borealis var. congolensis to species level: *Pinnularia congolensis* (Zanon) Cholnoky.

Zanon’s research was followed in the middle of the 20^th^ century by that of Hustedt (1949) who published a treatise on the diatoms of the “Parc national Albert”, nowadays the Virunga National Park, which was created in 1925 and among the first protected areas in Africa. Among the 55 new taxa that Hustedt (1949) described, 25 belong to the genus *Nitzschia* Hassall, 11 to *Navicula* Bory and 6 to *Eunotia*. The other taxa are from more than 10 other genera including *Achnanthes* Bory, *Amphora* Ehrenberg, *Caloneis* Cleve, *Cymbella*, *Fragilaria* Lyngbye, *Gomphonema* Ehrenberg, *Pinnularia*, *Stauroneis* Ehrenberg, *Stephanodiscus* Ehrenberg and *Surirella* Turpin (for details see Table 1).

Further studies which included diatoms, were carried out in the Kivu region in the 1950’s by Kufferath (1957). This author reported on diatoms from cataracts on the Rusizi River, which forms the overflow of Lake Kivu to Lake Tanganyika, near Bugarama. Although this village is located in Rwanda, formerly Ruanda-Urundi, we have included the new diatoms reported from the cataracts as the Rusizi River forms the border between DR Congo and Rwanda. Of the 59 taxa mentioned by Kufferath (1957), twelve were described as new to science (Table 1): five *Nitzschia*, two *Hantzschia* Grunow, two *Navicula*, one *Cymbellonitzschia* Hustedt, one *Gomphonema* and one *Pseudo-eunotia* Grunow. The other genera reported by Kufferath (1957) are *Amphora*, *Anomoeoneis* Pfitzer, *Cocconeis*, *Cymatopleura* W. Smith, *Cymbella*, *Gomphocymbella* O. Müller, *Mastogloia* (Thwaites) W. Smith, *Rhopalodia* O. Müller, *Rhoicosphenia* Grunow and *Synedra*.

At the beginning of the 21^st^ century research on algae of Lake Kivu continued with the work of Sarmento (2006) and Sarmento et al. (2012). Although not concerning the DR Congo, it is noteworthy to mention the papers of Mpawenayo (1985, 1996) as these concerned diatoms from rivers in the Burundian part of the Rusizi plain. The Rusizi River divides its plain in two parts, the river forming the border between DR Congo and Burundi.

We have not included the research conducted on diatoms of Lake Tanganyika because this ancient lake, located on the territory of four African countries (DR Congo, Burundi, Tanzania and Zambia), does not fall within the scope of this paper. Moreover, there are a number of reports regarding this lake which have been produced in the last decades (e.g. Cocquyt 1998, 2006).

Very little research has been done on freshwater algae from the remaining part of DR Congo (see also Taylor and Cocquyt 2015). For Kongo Central (called Bas-Congo in the colonial time and between 1997 and 2015, and Bas-Zaïre between 1965 and 1997) of note are the publications of Kufferath who reported eight taxa near Mateba Island (Kufferath 1956a) and 44 taxa, of which eleven species and two forms were new to science, at Banana Beach (Kufferath 1956b) (for details see Table 1): one *Coscinodiscus* Ehrenberg, one *Craspedodiscus* Ehrenberg, one *Melosira* C. Agardh, nine *Nitzschia* and one *Synedra*. It should be noted that some marine species are included in the results of these surveys, which is not surprising as the two localities are close to the mouth of the Congo River into the Atlantic Ocean. In 1948, Kufferath reported on the plankton of the Congo River near Makanza, formerly called New Antwerp, halfway between Kisangani and Kinshasa in the Equateur province. Among the 25 taxa Kufferath (1948) reported one new *Nitzschia* species (Table 1). He named this species after André Capart (1914–1991), who was the Director of the Royal Belgian Institute of Natural Sciences between 1958 and1978. Therefore the specific epithet should be written as *capartii*. In the second half of the 20^th^ century Cholnoky published three works on tropical African diatoms. Besides diatoms from Mount Kenya (Cholnoky 1960) and the Rwenzori mountains in Uganda (Cholnoky 1964), he studied the diatoms from the Bangweulu swamps (Cholnoky 1970) and reported on 92 specific and infraspecific taxa including three new species, one *Achnanthes*, one *Pinnularia* and one *Surirella* (Table 1). Although these swamps are located in Zambia we include these here as the swamps are situated in the upper Congo River basin and close to the border with DR Congo. Two decades later, Compère (1989) described a new *Stauroneis* from a fishpond in Kinshasa (Table 1).

The first investigation of the Tshopo province was carried out in the mid 1950’s (Demalsy 1957) on diatoms growing epiphytically on *Azolla* Lamarck (aquatic ferns of the family Salviniaceae) in the vicinity of Yangambi. The dominant genera were *Eunotia*, *Cocconeis* and *Achnanthes*; the other genera mentioned are: *Bacillaria* Ehrenberg, *Caloneis*, *Coscinodiscus* Ehrenberg, *Cyclotella* Kützing ex Brébisson, *Diatoma* Bory, *Diploneis* (Ehrenberg) Cleve, *Epithemia* Kützing, *Frustulia* Rabenhorst, *Gomphonema*, *Gyrosigma* Hassall, *Cymbella*, *Navicula*, *Nitzschia*, *Pleurostaurum* (Rabenhorst) C. Janisch, *Pinnularia*, *Stauroneis*, *Surirella*, *Synedra* and some other centric diatoms. Diatom research in the Tshopo province was started again at the end of the 20^th^ century, as is shown in the publication record. The Congo River as well as localities downstream the Lindi River, a major tributary of the Congo river, the Tshopo River and several small rivers and ponds in Kisangani were studied by Golama (1991, 1992, 1996), who reported 278 diatom taxa excluding desmids (group of green algae) and euglenophytes. In the same period a new *Gomphonema* species, *G. zairense* Compère, was described from the Tshopo River (Compère 1995) (Table 1).

Two decades later, renewed interest in diatom research in the region of Kisangani and Yangambi was initiated by the Boyekoli Ebale Congo 2010 expedition, an expedition that covered 250 km of the Congo River between Kisangani and Bumba and downstream some of its major tributaries (e.g. Cocquyt et al. 2013, 2016; Cocquyt and Taylor 2015). This research resulted in the description of several new diatom species belonging to the genera *Cavinula* D.G. Mann & Stickle, *Eunotia*, *Gomphonema*, *Navicula* and *Iconella* Jurilj (as *Surirella*) (for details see Table 1). *Cavinula lilandae* Cocquyt & M. de Haan (Cocquyt et al. 2013), a diatom from sandy substrata, for example, was described from a stream near the village of Lilanda located close to the western border of the Yangambi Biosphere Reserve. *Gomphonema grande* B. Karthick, Kociolek, J.C. Taylor & Cocquyt (Karthick et al. 2016) and *Navicula nielsfogedii* J.C. Taylor & Cocquyt (Taylor et al. 2016a) were described from an epiphytic sample taken in the Lomami River about 33 km as the crow flies from its confluence with the Congo River. This *N. nielsfogedii*, which may be conspecific with N. fuerbornii f. africana Foged described from Ghana (Foged 1966), has a distribution that is not restricted to the Congo, but to tropical and sub-tropical Africa (Taylor et al. 2016).The genus *Eunotia* is not only abundant in the acid streams and rivers from the Congo basin, but it is also a very diverse genus. Up to the present four new taxa have been described from the Yangambi Biosphere Reserve and its surroundings: *E. pierrefuseyi* (J.C. Taylor & Cocquyt) J.C. Taylor and Cocquyt, *E. leonardii* J.C. Taylor & Cocquyt, *E. rudis* Cocquyt & M. de Haan and *Geissleria lubiluensis* Cocquyt & Lokele (Table 1) (Cocquyt et al. 2016; Taylor et al. 2016b; Cocquyt and Lokele 2019; Taylor and Cocquyt 2019). Moreover, *Eunotia enigmatica* L.F. Costa & C.E. Wetzel a species recently described from the Amazon basin (Costa et al. 2017a, b) and another South American species, *Encyonopsis frequentis* Krammer (Krammer 1997) were observed in the Congo basin (Cocquyt et al. 2019).

However, what is not apparent from the above cited publications is that diatom research was also conducted in the region of Kisangani, Tshopo province, in the decades between the publication of the paper by Demalsy (1957) and those of Golama (1991, 1992, 1996). In the 1980’s several students completed their theses on diatoms at the University of Kisangani (UNIKIS): Golama in 1980, Dhed’a in 1981, Mbuyu and Mwilambwe in 1983, Kasereka, Kwere and Mbiya in 1984 (Table 2). The results of Golama on diatoms of the Lindi River and the Simi-Simi pond, and of Dhed’a, on diatoms of the Kabondo River and ponds near Botumbe, were published in the “Annales de la Faculté des Sciences de Kisangani”, the local journal of the University of Kisangani (Golama et al. 1983). A total of 21 genera were reported: *Asterionella* Hassall, *Caloneis*, *Ceratoneis* Ehrenberg, *Cocconeis*, *Coscinodiscus* (mentioned as *Cosnodiscus*), *Cylindrotheca* Rabenhorst, *Cymbella*, *Diatoma*, *Epithemia*, *Eunotia*, *Fragilaria*, *Frustulia*, *Gomphonema*, *Gyrosigma*, *Navicula*, *Nitzschia*, *Pinnularia*, *Rhopalodia*, *Surirella*, *Synedra*, *Tabellaria* Ehrenberg ex Kützing (Golama et al. 1983). The Lindi River and the Simi-Simi pond were the most diverse each with 16 genera; 7 genera were reported from ponds near Botumbe, 5 from the Kabondo River and 3 from a pond at Lumbulumbu.

**Table 2.** List of diatom-related theses authored by students in DR Congo with the academic year of submission, the student’s full name and affiliation (UNIKIS: Université de Kisangani; IFA: Institut Facultaire des Sciences Agronomiques de Yangambi; UOB: Université officielle de Bukavu), the academic degree and the title of the dissertation. A bachelor`s dissertation from the Thomas More University of Applied Sciences (Thomas More) in Belgium is added. A translation of the original French/Dutch title into English is given in italics. (*: not yet submitted).

Year	Institution	Full name	Degree	Title thesis
1980	UNIKIS	Anicet Golama Swana Kaketa	licentiate	Étude comparative de la flore algologique de la rivière Lindi et de l’étang de Simi-Simi (Haut-Zaïre) en relation avec quelques facteurs du milieu.
				*Comparative study of the algal flora of the Lindi river and the Simi-Simi pond (Upper Zaire) in relation with some environmental factors.*
1981	UNIKIS	Benoît Dhed’a Djailo	licenciate	Inventaire algologique des étangs de Botumbe et de la rivière Kabondo.
				*Algological inventory of the ponds of Botumbe and the Kabondo River.*
1983	UNIKIS	Mwilambwe Mbuyu Wa Kibwe	licenciate	Flore algale des réservoirs d’eau douce, étude des algues d’un étang à Kisangani.
				*Algal flora of freshwater reservoirs*, *study of algae of a pond in Kisangani.*
1984	UNIKIS	Mbiya Mutombo Mudima	licenciate	Contribution à l’étude de la flore algale d’une rivière de la sous-région urbaine de Kisangani: Makiso.
				*Contribution to the study of the algal flora of a river in the suburban region of Kisangani: Makiso.*
1984	UNIKIS	Kasereka Katswangene	licenciate	Contribution à l’étude de la flore algale d’une petite rivière de la sous-région urbaine de Kisangani: Djubudjubu I.
				*Contribution to the study of the algal flora of a small river in the suburban region of Kisangani: Djubudjubu I.*
1984	UNIKIS	Kwere Kwere Mughania	licenciate	Étude des algues des bassins d’épuration de l’usine de traitement des eaux à la Régideso Kisangani.
				*Study of the algae of the water purification basins of the water treatment plant at the Régideso Kisangani.*
2013	UNIKIS	Julienne Mukinzi Manyumba	licentiate	Contribution à l’étude des diatomées benthiques et périphytiques des quelques étangs de Ngene-Ngene aux environs de Kisangani (R.D. Congo).
				*Contribution to the study of benthic and periphytic diatoms of some ponds at Ngene-Ngene in the surrounding of Kisangani (DR Congo)*
2013	UNIKIS	Solange Mosunga Boamba	licentiate	Étude sur la composition des diatomées phytoplanctoniques des étangs de Ngene-Ngene situés en milieu périphérique de Kisangani.
				*Study on the composition of phytoplankton diatoms in ponds at Ngene-Ngene located in the peripheral area of Kisangani.*
2014	UOB	S. Ombeni	licentiate	Evaluation de la qualité de l’eau de la rivière Nyamuhinga (l’un des affluents Nord-Ouest du Lac Kivu) par les indices diatomiques. *Assessment of the water quality of the Nyamuhinga River (one of the northwestern tributaries of Lake Kivu) using diatom indices.*
2018	UOB	MwamiBantu Muliri Cédric-Dubois	bachelor	Diversité algale et caractéristiques physico-chimiques des eaux thermales de la rivière Mayi ya Moto, Nyangezi, Sud-Kivu.* Algal diversity and physico-chemical characteristics of the thermal waters of the Mayi ya Moto River, Nyangezi, South-Kivu.*
2019	UNIKIS	Alain Okito Mosindo	master	Étude des diatomées épiphytiques isolées des herbiers et plantes aquatiques fraîches de la région de Yangambi en République Démocratique du Congo (RDC).
				*Study of epiphytic diatoms isolated from herbarium materials and fresh aquatic plants collected in the region of Yangambi in the Democratic Republic of the Congo (DRC).*
2019	Thomas More	Zoë Madder	bachelor	Een onderzoek naar de evolutie van waterkwaliteit in de regio Eala, Kisangani en Yangambi (DR Congo) doorheen de 20^ste^ eeuw.
				*A study of the evolution of water quality in the Eala*, *Kisangani and Yangambi region (DR Congo) throughout the 20^th^ century.*
2019*	IFA	Nelly Asele Yapeti	bachelor	Identification des diatomées du cours d’eau Makiso dans la région de Kisangani en saison sèche et saison des pluies. *Identification of the diatoms of the Makiso watercourse in the region of Kisangani in the dry season and the rainy season.*
2019*	IFA	Francis Nzanzu Vosi	bachelor	La flore des diatomées du cours d’eau Lotuli dans la région de Yangambi en saison sèche et saison des pluies.
				*The diatom flora of the Lotuli River in the region of Yangambi during the dry season and the rainy season.*
2019*	IFA	Daniel Mabele Boyoma	bachelor	Identification des diatomées du cours d’eau Loile dans la région de Yangambi en saison sèche et saison des pluies.
				*Identification of diatoms from the Loile River in the Yangambi region in the dry and the rainy season.*
2019*	UNIKIS	Dorcas Basuma Sakina	licentiate	Identification des diatomées du cours d’eau Lokwae dans la région de Kisangani en saison sèche et saison des pluies.
				*Identification of the diatoms of the Lokwae watercourse in the region of Kisangani in the dry season and the rainy season.*
2019*	UNIKIS	Anastasie Batchangondua Beyanga	licentiate	Influence de la saison sur la flore diatomique d’un cours d’eau. Cas de la rivière Masindula dans la région de Yangambi.
				*Influence of the season on the diatom flora of a watercourse. Case of the Masindula River in the Yangambi region.*
2019*	UNIKIS	Jean Claude Makambo Tindya	licentiate	Influence de la saison sur la flore diatomique d’un cours d’eau. Cas de la rivière Lokombe dans la région de Yangambi.
				*Influence of the season on the diatom flora of a watercourse. Case of the Lokombe River*, *in the region of Yangambi.*
2019*	UNIKIS	Bienfait Nzanzu Vivuya	licentiate	Influence de la saison sur la flore diatomique d’un cours d’eau. Cas de la rivière Losa dans la région de Yangambi.
				*Influence of the season on the diatom flora of a watercourse. Case of the Losa River in the region of Yangambi*.
2019*	UNIKIS	Jules Abani Sifa Zolianse	master	Analyse de l’impact de l’anthropisation et saisonnier sur la diversité de diatomées dans la rivière Kabondo (Province de la Tshopo, R.D. Congo).
				*Analysis of the human impact and seasonality on the diversity of diatoms in the Kabondo River (Tshopo Province*, *DR Congo).*
2019*	UNIKIS	Nathalie Longonya	master	Variations spatio-saisonnière et l’influence des activités anthropiques sur le développement de peuplement algale (diatomées) de la rivière Yoko 1 à la Réserve Forestière de la Yoko.
				*Spatio-seasonal variations and the influence of human activities on the development of algal (diatoms) communities of the Yoko 1 River at the Yoko Forest Reserve.*
2019*	UNIKIS	Marie-Claire Lissasi Songowali	master	Variations spatio-saisonnière et l’influence des activités anthropiques sur le développement de peuplement algale (diatomées) de la rivière Yoko 2 à la Réserve Forestière de la Yoko.
				*Spatio-seasonal variations and the influence of human activities on the development of algal (diatoms) communities of the Yoko 2 River at the Yoko Forest Reserve.*
2020*	UNIKIS	Edit Lokele Ndjombo	PhD	Etude des diatomées de quelques cours d’eau de Yangambi, dans le district de la Tshopo.
				*Study on diatoms from some rivers at Yangambi*, *Thsopo district*.

A pond (étang du Grand-séminaire) in Kisangani, dominated by *Closterium* Nitzsch ex Ralfs (Desmidiales), was also investigated. It is located 4.5 km from the old road to Buta in the north of the city. Mbuyu (Table 2) reported twelve diatom genera in samples from the dry and the wet season in 1983. *Surirella* was only observed in the wet season, while *Amphipleura* Kützing, *Cymbella*, *Epithemia*, *Fragilaria*, *Melosira*, *Navicula*, *Nitzschia*, *Pinnularia*, *Rhoicosphenia*, *Synedra* and *Tabellaria* were observed both in the dry and the wet season. Kasereka (Table 2) studied the algal flora of the Djubudjubu River, where he mentioned the following diatom genera from 27 samples taken between 10 March and 3 May 1984: *Asterionella*, *Nitzschia* in the plankton, *Fragilaria*, *Gomphonema*, *Navicula* and *Pinnularia* in the benthos, *Cocconeis*, *Cymbella*, *Fragilaria*, *Gomphonema*, *Navicula*, *Nitzschia*, *Pinnularia*, *Surirella* and *Synedra* in the epilithon. Diatoms of the genera *Gomphonema* and *Navicula* were the most abundant. Kwere (Table 2) reported on algae present in the purification ponds of the water treatment plant of the Régideso in Kisangani. Seven diatom genera were mentioned, *Navicula*, *Nitzschia* and *Pinnularia* were dominant, *Asterionella*, *Fragilaria*, *Gomphonema*, *Melosira*, *Surirella* and *Synedra* were also recorded. Mbiya (Table 2) studied the algal flora of the Makiso River in the urban subregion of Kisangani. In epilithic samples eight genera were reported (*Cocconeis*, *Cymbella*, *Gomphonema*, *Navicula*, *Nitzschia*, *Pinnularia*, *Surirella* and *Synedra*) and only four were found in the benthos (*Navicula*, *Nitzschia*, *Pinnularia*, and *Synedra*).

Golama and Richell-Maurer (1983) reported on fish stomach contents from several fish species captured in the Lindi and the Congo River near Kisangani. *Melosira* was found to be dominant in the stomach contents of *Citharinus* sp., a tropical African lutefish, and *Labeo* sp., a genus of carp, while *Cymbella* and *Navicula* were found in *Distichodus* sp., an African ray-finned fish. In addition to diatoms belonging to these three genera, 15 other genera were reported: *Amphora*, *Amphipleura*, *Arcella* Ehrenberg, *Cocconeis*, *Caloneis*, *Cymatopleura*, *Cylindrotheca*, *Diatoma*, *Fragilaria*, *Gomphonema*, *Gyrosigma*, *Nitzschia*, *Pinnularia*, *Surirella* and *Synedra*.

The Boyekoli Ebale Congo 2010 expedition, together with initiatives taken by the VLIR–UOS at the University of Kisangani and the FORETS project at Yangambi, encouraged a number of students to choose diatom related subjects for their theses. In 2013, two students investigated the diatoms of some fish ponds at NgeneNgene, about 20 km from the city center of Kisangani. One thesis concentrated on the diatoms in the phytoplankton (Mosunga), the other on the benthos and the epiphyton (Mukinzi) (Table 2). These two students tried to delineate taxa within diatom genera. However, as the available literature or diatom floras for tropical Africa were scarce or not available to the students, a name could not be given to most of the taxa. In the phytoplankton samples 27 taxa were reported belonging to *Asterionella*, *Aulacoseira* Thwaites, *Cyclotella*, *Cymbella*, *Encyonema* Kützing, *Eunotia*, *Fragilariforma* D.M. Williams & Round, *Frustulia*, *Gomphonema*, *Navicula*, *Nitzschia*, *Pinnularia*, *Sellaphora* Mereschkowsky, *Stenopterobia* (Brébisson) Van Heurck and *Surirella*. For the benthos and epiphyton a total of 13 taxa were reported (12 for the benthos, 9 epiphytic on *Nymphaea lotus* L. and 6 epiphytic on *Azolla pinnata* R. Brown). Most were the same genera as reported in the plankton, however *Asterionella* and *Cymbella* were not present in the periphytic samples while *Cymatopleura* was present but not in the plankton.

Okito studied diatoms present on herbarium material of aquatic plants collected during the 20^th^ century in the Central Forest phytogeographic region (VI) according to the classification of Robyns (Robyns 1948; Bamps 1968) and kept at the Herbarium of Yangambi (YBI) (Table 2). In a similar fashion to the students Mosunga and Mukinzi, Okito tried to distinguish the different species, without, however, giving a name to most of them. This resulted in 104 specific and infra specific taxa, belonging to 34 genera with *Eunotia*, *Frustulia* and *Desmogonium* Ehrenberg as most dominant. The other observed genera were *Achnanthes*, *Achnanthidium* Kützing, *Actinella* F.W. Lewis, *Amphora*, *Aulacoseira*, *Bacillaria*, *Brachysira* Kützing, *Caloneis*, *Cavinula*, *Cocconeis*, *Cyclotella*, *Cymbopleura* (Krammer) Krammer, *Diploneis*, *Encyonema*, *Encyonopsis* Krammer, *Fragilaria*, *Fragilariforma*, *Frustulia*, *Gomphonema*, *Luticola* D.G. Mann, *Neidium*, *Nitzschia*, *Orthoseira* Thwaites, *Placoneis* Mereschkowsky, *Planothidium* Round & Bukhtiyarova, *Pinnularia*, *Sellaphora*, *Stauroneis*, *Stenopterobia*, *Surirella* and *Ulnaria* (Kützing) Compère. A professional bachelor thesis at the Thomas More University of Applied Sciences, Geel, Belgium, was written by Madden on epiphytic diatoms growing on a restricted number of *Nymphaea lotus* herbarium specimens from the same phytogeographic region (VI) (Table 2). The sampled herbarium specimens are from the collections of the herbarium of the Meise Botanic Garden (BR). This student reported on about 180 taxa belonging to 42 genera. In addition to the genera given by Okito, she also observed *Craticula* Grunow, *Diadesmis* Kützing, *Eolimna* Lange-Bertalot & W. Schiller, *Fallacia* A.J. Stickle & D.G. Mann, cf. *Fistulifera* Lange-Bertalot, *Geissleria* Lange-Bertalot & Metzeltin, *Halamphora* (Cleve) Levkov, *Humidophila* (Lange-Bertalot & Werum) Lowe, Kociolek, Johansen, Van de Vijver, Lange-Bertalot & Kopalová, *Iconella*, *Mayamaea* Lange-Bertalot, *Navicula*, *Nupela* Vyverman & Compère, *Staurosira* Ehrenberg, *Staurosirella* D.M. Williams & Round.

During the academic year 2017–2018 several other students started studying diatoms in rivers in the Tshopo province. Although most are not finished at the time of publication of the present paper, the preliminary titles of these theses (bachelor, licentiate or master level) are included in Table 2.

Algological investigations, other than on Lake Kivu, continue in the South Kivu province through student theses (Table 2). Muliri (Table 2) for example reported on 18 diatom genera from the thermal water of the Mayi ya Moto River. From the genera cited (*Achnanthes*, *Actinella*, *Aulacoseira*, *Bacillaria*, *Cocconeis*, *Cyclotella*, *Diadesmis*, *Diatoma*, *Encyonopsis*, *Fragilaria*, *Fragilariforma*, *Melosira*, *Navicula*, *Nitzschia*, *Stephanodiscus*, *Synedra*, *Tabellaria* and *Thalassiosira* Cleve) we can conclude that more recent literature (e.g., Round et al. 1990, and subsequent later taxonomic publications) is already being used. For example the genera *Diadesmis* and *Encyonopsis* are used which were before lumped with the genera *Navicula* and *Cymbella* respectively.

**Figure 1.**
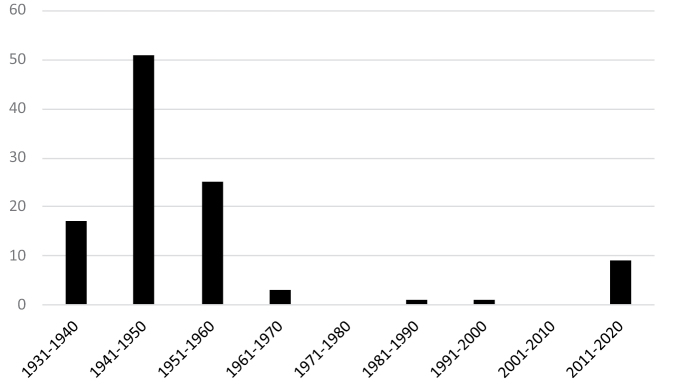
Number of new diatom taxa (specific and infraspecific) described from DR Congo per decade.

**Figure 2.**
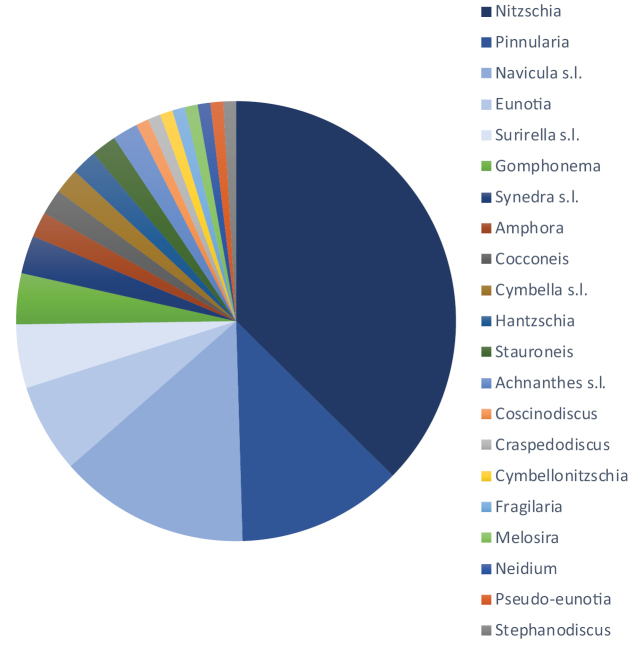
Pie diagram showing the relative abundance of the 21 genera (s.l.) to which the newly described species and infraspecific taxa from the DR Congo belong, *Nitzschia* being the most important, followed by *Pinnularia* and *Navicula* s.l.

Up to now a total of 106 new diatoms (specific and infraspecific taxa) have been described from DR Congo, with a peak (51 taxa) at the end of the 1940’s (Fig. 1). Of the 21 genera (s.l.), *Nitzschia* is by far the genus with the highest numbers of new taxa described from DR Congo (40), followed by *Pinnularia* (12) (Fig. 2). *Navicula* s.l. has 15 taxa, but includes at least two *Craticula*, one *Cavicula*, one *Geissleria*, one *Luticola* and one *Mayamaea* species. Although the genus *Eunotia* is well represented in the acid rivers of DR Congo, it only comes in fourth place with 7 new species described. However, the renewed interest in the diatom biodiversity in DR Congo will certainly increase the number of new diatom species to be discovered, including several *Eunotia* as evidenced by ongoing investigations (unpubl. data). Of interest are the similarities and differences with the neo-tropical (South America) diatom flora as evidenced by the presence of *Eunotia enigmatica* L.F. Costa & C.E. Wetzel and *Encyonopsis frequentis* Krammer (Cocquyt et al. 2019) in DR Congo.

It is worth noting that almost all of the new diatoms (see Table 1) described from DR Congo have the taxon status uncertain (unassessed) in DiatomBase. Only twelve taxa have the taxon status accepted; these include nine species described from DR Congo in the 21^st^ century (Cocquyt et al. 2013; Cocquyt and Taylor 2015; Cocquyt et al. 2016; Karthick et al. 2016; Taylor et al. 2016a, b; Cocquyt and Lokele 2019) as well as *Eunotia pseudoflexuosa* Hustedt, *Nitzschia elliptica* Hustedt and *Pinnularia tropica* Hustedt. All three aforementioned species were described in a publication in which Hustedt described a total of 50 new taxa from the “Parc national Albert” (Hustedt 1949). Although *Nitzschia epiphyticoides* Hustedt was thoroughly studied (Cocquyt et al. 2012) it still has the status uncertain (unassessed) in DiatomBase (Kociolek et al. 2019).
